# Supplementary material for: Autocatalytic activation of a malarial egress protease is druggable and requires a protein cofactor
Source: EMBO J. 2021 May 1;40(11):e107226. doi: 10.15252/embj.2020107226 (PMC8167364; doi:10.15252/embj.2020107226)
Supplement: Supplementary file 1 — Appendix [file EMBJ-40-e107226-s001.pdf]

Index of Appendix Figures

1. Appendix Figure S1: E64-d is a more potent inhibitor of SERA6 maturation than E64.
2. Appendix Figure S2: MSA180 contains three distinct conserved segments.

Appendix Figure S1. E64-d is a more potent inhibitor of SERA6 maturation than E64.

(A) Western blot comparing maturation of SERA6-mTAP from *SERA6-mTAP:loxP* parasites in the presence of E64 and E64-d (reproducible in 2 independent experiments). (B) Western blot showing cleavage of host RBC β-spectrin (red arrowhead) is inhibited by both inhibitors.

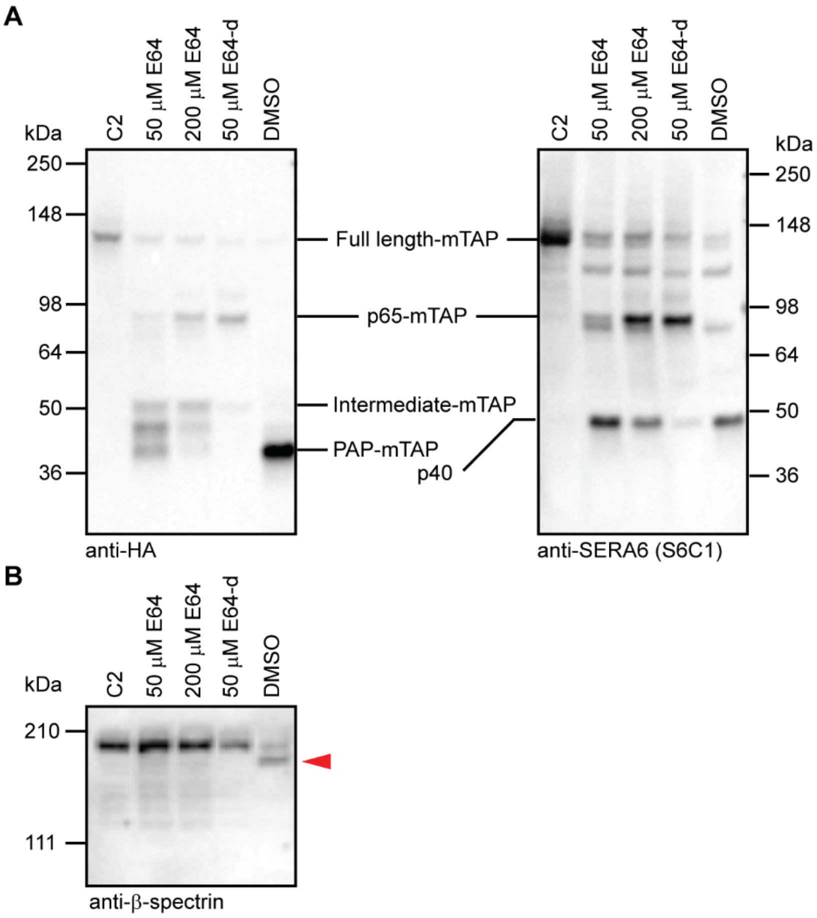

Appendix Figure S2. MSA180 contains three distinct conserved segments.

Alignment of predicted primary sequences of MSA180 with orthologues from other human-infecting *Plasmodium* species. The secretory signal peptide is shaded grey and the two non-contiguous segments of the *P. falciparum* protein found to co-precipitate with SERA6 PAP-mTAP are indicated by red lines. Sequences used (as synthetic peptides) to raise polyclonal antibodies to MSA180 p40 and p45 are highlighted. The N-terminus of a semi-tryptic peptide likely corresponding to the N-terminus of p45 is indicated (red triangle). This was shown to be a PfSUB1 cleavage site (Fig EV4).

```
P. falciparum      MNRIFYFCLFTILFWLSLVSGENVNNKNCNEKNRKAILLALLKNSLVDNKDYNNSEELKY    60
P. malariae        MLRIAYFSLSFIILSFLFFSGHNAL-PNEEDKNKKAILLALLTNTFINNKEYKNGEDINI    59
P. ovale           MLRIIYFSFFPILFSLFLISGHDAI-SNVEDKTKKAILLALLKNTFIDNEEYKEPNDLNN    59
P. knowlesi        MSRITFLFSLSILFFFFLLPGQNAL-TIDDDKNKRATLLALLKNTFIDNKGNKKSDDIKG    59
P. vivax (Sal-1)   MPRITPLFLLSILLSFFLFSGQNAL-TNDDDTNKRATLLALLKNTFIDNTENKKPDDINT    59
P. vivax (P01)     MPRITPLFLLSILLSFFLFSGQNAL-TNDDDTNKRATLLALLKNTFIDNTENKKPDDINT    59
                   * **  :    *:: : :. *.:.     ::..::* *****.*::::*   :: ::::

P. falciparum      ALEHIQNSELYPKDSKKFDKFIDEFFSYYNIHVNFTDEEKRILHISGVFKEFYVDVDNLN    120
P. malariae        ALENINNMKLHPTDNDKFDKFLDALFKHHNIYVTLDDHDKRIIHISGVLSEFYVDVDTLT    119
P. ovale           ALENINNMNIHPTDNKKFDNFLEELFKHYNVHVTFSDMDKRILHTSGVFNDIYVDVNSLD    119
P. knowlesi        ALENIKNMTLQPTDTDKFDKFLDQFLKFFQIYVTFSDKDKRVLHLSGVLNDVYVDVDSLS    119
P. vivax (Sal-1)   ALENINNMTLHPTDTDKFNKFLDHFLKFFHIYVSFSDKDKRVLHLSGVLNEVYVDVESLS    119
P. vivax (P01)     ALENINNMTLHPTDTDKFNKFLDHFLKFFHIYVSFSDKDKRVLHLSGVLNEVYVDVESLS    119
                   ***:*:*  : *.*..**::*:: ::...:::*.: * :**::* ***:.:.****:.*

P. falciparum      KDEMKEYFKKNYEKGLSLINLIVHSNLIIQQFDHDIIDKKKVH-----------------    163
P. malariae        EEKQKEYFNTFYEKGHTLINLILHSNLIHTKHDKDVNKENHKEETKPNHPDSPIPDEEPN    179
P. ovale           KGNTKEYFNGIHKKALSLINLVLHSNLVHPKYAEINIEKGSQTGNDLIQENSQ------D    173
P. knowlesi        KENLQKHFDSLYEKGLNLINLIVHSNLVHPKYDETEMHGVDAEEKDH-------------    166
P. vivax (Sal-1)   EENLQKHFDSVHEKGLNLINLIVHSNLVHPKYDETAVQGMEGEEPEQ-------------    166
P. vivax (P01)     EENLQKHFDSVHEKGLNLINLIVHSNLVHPKYDETAVQGMEGEEPEQ-------------    166
                   : : :::*.  ::*. .****::****:  :. .   .

P. falciparum      ------------------------------------------------------------    163
P. malariae        LEKEPIPDDVSKGEVNHQLNYNNVYDSDIEVESNHEVEPNHEVEPNHAVEPNHAVEPNHA    239
P. ovale           TE-GEPKGNTGEG------------------HE-----PVYDFKNNQGGEPDNAYGHIQG    209
P. knowlesi        VS----------------------------------------------------------    168
P. vivax (Sal-1)   TD-GPTEGPGSHGKV----------------DPPQEGDPQEEEGPSQEGHPQEKVDPTQE    209
P. vivax (P01)     TD-GPTEGPGSHGKV----------------DPPQEGDPQEEEGPSQEGHPQEKVDPTQE    209


P. falciparum      ------------------------------------------------------------    163
P. malariae        VEPNHAVEPNHATEPNHATEPNPENEPIPDDVSKGEVNHQLNYNNVYDSDIEVESNHEVE    299
P. ovale           EEPVHEYEYNQGGEPDNACEHIQGEEPVH---E-------YEYN----------------    243
P. knowlesi        -----------------DNYAENIKGPAHYEES-------QEYE----------------    188
P. vivax (Sal-1)   NHPHEKVDPPQEGDPAQESHPHQKDGPAQQDHS-------QEYA----------------    246
P. vivax (P01)     NHPHEKVDPPQEGDPAQESHPHQKDGPAQQDHS-------QEYA----------------    246


P. falciparum      ------------------------------------------------------------    163
P. malariae        PNHEVEPNHAVEPNHAVEPNHATEPNHATEPNHATEPNHAVEPNHATEPNPENEPNPENE    359
P. ovale           --------------------------------QGEEPV----------------------    249
P. knowlesi        --------------------------------EMPEYA----------------------    194
P. vivax (Sal-1)   --------------------------------VTPEYA----------------------    252
P. vivax (P01)     --------------------------------VTPEYA----------------------    252


P. falciparum      ------------------------------------------------------------    163
P. malariae        PIPDDVSKGEVNHQLNYNNVYDSDIEVEPNHAAEPNPENEPIPDDVSKGE-VNHQLNYNN    418
P. ovale           ------------HEYEYNQG------EEP-----------VHEYEYSQGEEPVHEYEYNQ    280
P. knowlesi        ------------DHYHYGGN------KED-----------PDDMDYENGEEYDAQKYQDD    225
P. vivax (Sal-1)   ------------DHYHYGGH------EED-----------PEDMDYENGEEYDAQGDPDD    283
P. vivax (P01)     ------------DHYHYGGH------EED-----------PEDMDYENGEEYDAQGDPDD    283
```

```
P. falciparum        ------------------------------------------------------------   163
P. malariae          VYGSDHEDETNHETEFKQEETHNYEYEAKQEDEHKEHYEHSHTNEPEKEVAHSYEAEHKK   478
P. ovale             G------EEPVHEYEYNQGEEPVHEYEHNQEGESDNAYEL-------------YHGGD--   319
P. knowlesi          H------YHHDEDYEYEEQDHANHDNHNDHDDHNDHDDHN-------------DHDDHND   266
P. vivax (Sal-1)     H---------DEHYEFDEHDE---------------------------------HGEH--   299
P. vivax (P01)       H---------DEHYEFDEHDE---------------------------------H-----   296


P. falciparum        ----------------------EQNTNTNKTLEYISDNLNDLINFKNIHLNNNSTGDFII   201
P. malariae          EDKPMQENEENYQY--GDDQEYDEEEFKNKNVEYIQKKLKNLLAMKSIKLNNNSNGEFKV   536
P. ovale             ------ITEEYYDNEGH-----NEENYKNKNIKYVGKKLKNLLAMKNIKLNNNSTGEFKV   368
P. knowlesi          HDDH-NDHNDHNDHDDHNDHNDHDDEEEKQKIKYVGKKLKNLLAMKNIKLNNNSTGEFKV   325
P. vivax (Sal-1)     ---------GDYDHDEHGEHDEPYHEEDKKKIKYVGKKLKNLLAMKNIKLNNNSTGEFKV   350
P. vivax (P01)       --------------DEHGEHDEPYHEEDKKKIKYVGKKLKNLLAMKNIKLNNNSTGEFKV   342
                                             .   ::.::*: .:*::*: :*.*:*****.*:* :

P. falciparum        KLYTNYVNYIN-PYQTNPLPNTPHYYEHKNFHTKEHYI-------YDEEIVNPNMDNINT   253
P. malariae          NFYTNYVNYINQPPSNS-GSNHQEKYEYTEINQESKKKKKKS-----------------D   578
P. ovale             NFYTNYVNYIS-PYSINPFPSSQDMHKDNKVYEEGRNGKEEKYDEYSGGVSDGDK--DDS   425
P. knowlesi          NFYTNYMNYINTPYVEPPFPFHKDIYEYAEVYSGDKIYPKD-------------------   366
P. vivax (Sal-1)     NFYTNYVNYINTPYGAPLLPFHKDSYEYAEVYSGDKLHPKK-------------------   391
P. vivax (P01)       NFYTNYVNYINTPYGAPLLPFHKDSYEYAEVYSGDKLHPKK-------------------   383
                     ::****:***. *          . ::  :.    :

P. falciparum        HTEEDNVYV-------SATKGNQKEETEK-KENHENNNAINPKYMN--------------   291
P. malariae          KMDYNSYFVKEVPTVVGEDEG--------EGDDIVNTNDIKQVNENLYNTNKAYDHMYNK   630
P. ovale             SGSHGGYYAGK-------------EEGETEDGYNKYRNAIRNIYEKMNNQSE-EEHEYEE   471
P. knowlesi          HKDDNIYYGGENELIPQHVKGEMQKEALQEGSYHGYKSTMKGMYENIKKRSG-KK-----   420
P. vivax (Sal-1)     HGDEQMYYVGEKELIPVHGKGDMQK----EGPYDVYKGAMKGIYENIKKKAA-KK-----   441
P. vivax (P01)       HGDEQMYYVGEKELIPVHGKGDMQK----EGPYDVYKGAMKGIYENIKKKAA-KK-----   433
                       .    :                             . :.    :

P. falciparum        --------------YETYYKKIFNAIFEQIDKLNKTLFEIKNKNNSETNEN---------   328
P. malariae          K------IEGMEEKANKYYPYDDSS-----------EYA--------DNARYLKKGN---   662
P. ovale             NAGEEQGKEGNKEWERDWYKDENSE-----------G------ERCKRDEHFV-------   507
P. knowlesi          ------------ECKSGWCGKKMAG-----------KYQMMHDNNCNNDENNDG------   451
P. vivax (Sal-1)     ------------GGKNGWWGKKKAG-----------KYQIKEDSNGDDDDDEDGDGDGDD   478
P. vivax (P01)       ------------GGKNGWWGKKKAG-----------KYQIKEDSNGDDDDDEDGDGDGDD   470
                                      :                              :

P. falciparum        ------------------------------------ISESNSGNPELNNENSYSVKLSSS   352
P. malariae          ----------------ELNKEGNNYYYYDDGKTQHRRSN----EYEQDRGNINGMKMSNK   702
P. ovale             ---------------EEETEYAD-SYFDGEDDV-NRKYTEEEQTEYSQKDDVEGMQLGNK   550
P. knowlesi          ---------NDHDDHDEDDQLQNELLHQGHGTKKKSKCAHN--NKKLHGQNVDDKEITDK   500
P. vivax (Sal-1)     DDDDDNGDDDDDEDPDEDDQLQNEPLHKGHGPNKKPKYGHK--KKKIHGENVDDEETTDK   536
P. vivax (P01)       DDDDDNGDDDDDEDPDEDDQLQNEPLHKGHGPNKKPKYGHK--KKKIHGENVDDEETTDK   528
                                                                    .  : . :  ..

P. falciparum        SPNSTNKESLIFPYTYYNPYYMFRLTNNFKENDEGLK-------NENNI----------N   395
P. malariae          KPGKSEAKASHFPYTYYNPYYMYSLNSTGSPKYNSNSKYNNGYTNGHNNEYNYEYSNKYN   762
P. ovale             -SPTGEKKGLNFSYTFYNPYYMYRLGSKIPDNEKINK-NDKSYHIVKK------------   596
P. knowlesi          APAEKEKKGIDFSYTYHNPYYMFKLGSSMPTGK-------------GGH---------LK   538
P. vivax (Sal-1)     APSEKGKKGIDFAYTYYNPYYMFKLGSNMPTGKKAQP-SGKGAPAKGGL---------LK   586
P. vivax (P01)       APSEKGKKGIDFAYTYYNPYYMFKLGSNMPTGKKAQP-SGKGAPAKGGL---------LK   578
                            :.  * **::*****: * ..
```

```
P. falciparum        -------------------NN--EDNQNDNMN----------------------------     406
P. malariae          DKYNDKYNNKYSNKYSNKYNNEYNNEYNNEYNNEYNNKYNNEYNNKYNNKYNNEYNNKYN     822
P. ovale             -----------------------QEKLVGE------EKWK--------------------     607
P. knowlesi          ---------------GGKDDKEEEEEEVDD------------------------------     553
P. vivax (Sal-1)     ---------------GGKGHDEEEEEVADE------EEEEEE-E--------A-------     609
P. vivax (P01)       ---------------GGKGHDEEEEEVADE------EEEEEEEA--------E-------     602
                                            :::  .:

P. falciparum        -------------I-------------------------------------VLGKIHNIL     416
P. malariae          NEYNNKYNNKYNNKYSDKYSDRYKKYNNNYYNNYNKKQNKKSLYDFMLQKKALEKE-HML     881
P. ovale             --------------------DS--------------------------------------     609
P. knowlesi          ------------------------------------------------------------     553
P. vivax (Sal-1)     -------------EVA----DAE------------------DVADEEDAEEVADED-ADV     633
P. vivax (P01)       -------------EVA----DAE------------------DVADEEDAEEVADED-ADV     626


P. falciparum        KDFNINENIMTNKMSAPLIMTIILNFFKKYMAENKFNLPMSSEVENKINKSNNKALLQQS     476
P. malariae          KNNYENQESNTKKNKIGLFLDTLTE-LARYIESTINKNKINASKMKNNQLGDNKHDAIQQ     940
P. ovale             --TSIDYENITNKNSVEYFFNTLLE-LADYVAKNMNKSNLSGNGKKK-KHLGDQHVLLQN     665
P. knowlesi          --ENGMEKNIEGESGANTFVNTLLE-LEGYVQPKESEH--------S-ELKEDKNTLGQS     601
P. vivax (Sal-1)     TDGDDAEKQHAKKSAANLFVNTLLE-LAGYLEPSESKK--------S-ELKEDKNSLGQA     683
P. vivax (P01)       TDGDDAEKQHAKKSAANLFVNTLLE-LAGYLEPSESKK--------S-ELKEDKNSLGQA     676
                            :    :     :.  : : :  *:  .  :         . :   ::    *

P. falciparum        NKDTPIHKKKEIRNKKIQTKVDVIDEKTKKKIANTIYVNVGQSGINGFFNFFDFREKSID     536
P. malariae          SKKRPIHKRKEMKSRKMKTKTDLVDEKEKEKIQDTMFVKIGQNGTIGLLNFFDFREGLLK     1000
P. ovale             GKNRPIYKKKEMKSKKMKTKKDEIDENMVNKIKDTMYFKVGQNGTNNFLNFFDFREDTLK     725
P. knowlesi          NKNRPIYKKKEMKSRKMKTKKDIPDEKITEKVKDTMYVKVGQNGTNGFLNFFDFREHSLK     661
P. vivax (Sal-1)     NKSRPIYKKKEMKSRKMKTKKDIVDEKTTEKIKDTMYVKVGQNGTNGFLNFFDFREYAVK     743
P. vivax (P01)       NKSRPIYKKKEMKSRKMKTKKDIVDEKTTEKIKDTMYVKVGQNGTNGFLNFFDFREYAVK     736
                     .*. **:*:**::.:*::** *  **:  :*: :*::.::**.*  .::*******  :.

P. falciparum        SNIFDLLHVMEDMKIFDIFQTIIFIQKFTENVCASYCMNITDVLELSHYDMIFYDKMVFH     596
P. malariae          NNFKDLIYVMEYLNVFNITETIMFIQKFTESICASYCMGITNVLELSNNDMLLYEKMRLI     1060
P. ovale             KNFTDLLLIMEQLKVFTISETIMFIQKFTETICASYCMGITDVLELSNNDMLLYEKMSIH     785
P. knowlesi          KNFEDLLKVMEFLKVFNITETIIFIEKFTQSVCASYCMGITDVLELVNNDMLLYEKMSFH     721
P. vivax (Sal-1)     ENFKDLLKVMETLKVFNITETIIFIQKFTESVCASYCMGITDVLELVNNDMLLYEKMSFH     803
P. vivax (P01)       ENFKDLLKVMETLKVFNITETIIFIQKFTESVCASYCMGITDVLELVNNDMLLYEKMSFH     796
                     .*: **: :** :::* * :**:**:***:.:******.**:**** : **::*:** :

P. falciparum        FSKDGMMIKT-DKKYLYNLKEFENILNLLNINANTIALNCTCKFYVDVNYTYSEQYKMHL     655
P. malariae          FNNQGMTVVTKNSEYEFNDAEFEKFLLLLNLNKYTIPLNCPCKFYTNNIISYYIQYNSGL     1120
P. ovale             FDSKGMTVILENTEHHFSDTEFERILNLLNINKDTIPLTCPCKFYTNSIISYCKQYKSSL     845
P. knowlesi          FHKNGMIVTT-NSNYEFNGIEFESLLDLLNINKETIPLICPCKSYTNNIISYCQQYKYNM     780
P. vivax (Sal-1)     FRKNGMTVTT-NSNYEFNGINFESLLALLNINKETIPLTCPCKSYTNNIISYCKQYKSNL     862
P. vivax (P01)       FRKNGMTVTT-NSNYEFNGINFESLLALLNINKETIPLTCPCKSYTNNIISYCKQYKSNL     855
                     * ..** :   :.:: :.  :** :* ***:*  ** * * ** *.:   :*  **:  :

P. falciparum        KGYLHKMNEFDYINNFSASYLLNQLIIFQDKFNYIKMNGKLPIDDPKNI---YNMNNVHD     712
P. malariae          KGNFYQLKKSQFTEKFSPSYLLDKLIVLQDKLNYIRKHGKLEVNKRAVKNEDDDDDDFHE     1180
P. ovale             KGYFEQSKKAEYINKFSISYILQQLENLQEKLYYIKKNGKLECKQSSKC---YDGNDLYN     902
P. knowlesi          KGYFTQSNNSEYLEKFDAYYLLEDLILLEEKLNDIKKNGKISSDKFVNI---LDSKDLYT     837
P. vivax (Sal-1)     KGYFTQSKNSEYLEKFTPYYLLEQLILLEDKLNYIKKNGKISSDTSVKI---LESKDLHT     919
P. vivax (P01)       KGYFTQSKNSEYLEKFTPYYLLEQLILLEDKLNYIKKNGKISSDTSVKN---LESKDLHT     912
                     ** : : :: :: ::*   *:*:.*  :::*:  *: :**:  .         : .:.:
```

```
P. falciparum        TAYYHNSRYFPTKDMPSLE-----------------------------------------DN    733
P. malariae          NVYYHKQRYIPTIRTLNVENNSNT-----------------------------------TDN    1207
P. ovale             TAYYHNERYFPTITTLNVSDGCSNGND-GSDGSDRSDGSGGSGGS-DGSDGSGDEGNQVK    960
P. knowlesi          TAYYHNSRYFPPLRTCSASTTAPVINVSDKSAPNLASGFGEKVVDEIGLSKHGSNKAGVA    897
P. vivax (Sal-1)     TAYYHNNRYFPPLKASSASSTAPVSAVGGKSGLNRVGGFGGNGVDGNGVSSHGSNHGGGE    979
P. vivax (P01)       TAYYHNNRYFPPLKASSASSTAPVSAVGGKSGLNRVGGFGGNGVDGNGVSSHGSNHGGGE    972
                     ..***:.**:*     . .

P. falciparum        FYEHLKYPDINTIHIYYNASPVKLNEVNDLKTIIIDEIKSKIFYINSYRVGDQFFPTYSN    793
P. malariae          TVNNFTYPDMDLLIVYYNSPLVNFKNLTDVKNILIEEVNSKIFYINSFRIGNQFFPTYSN    1267
P. ovale             TEVHLKYPDVETINVYYNASPVNLKNVNDVKNVLIEEVKSKIFYINSYRIGNQFFHTYSN    1020
P. knowlesi          TGDKLTYPDVDALNIYYNASPVNLKNIQDVKSVLIDEVKSKIFYINSYRIGNQFFPTYSN    957
P. vivax (Sal-1)     LADELKYPDVDTLNIYYNASPVNLKSIHDVKNVLIDEVKSKIFYINSYRIGNQFFPTYSN    1039
P. vivax (P01)       LADELKYPDVDTLNIYYNASPVNLKSIHDVKNVLIDEVKSKIFYINSYRIGNQFFPTYSN    1032
                        .:.***:: : :***:  *:::.: *:*.::*:*::********:*:*:*** ****

P. falciparum        LGKDDHDLEHSAKNFYNISNENGDNTFNNNNNNMDNKKRMYNYNKHKDNDSRYTDNSNKN    853
P. malariae          LGKDDHDLEILEEVNASKINAPSRN-----------------------------------    1292
P. ovale             FGKDDHDLEIMEAVNTSKSKGENNS-----------------------------------    1045
P. knowlesi          QGKDDHDLEIMQSDNSSKMNVEDKT-----------------------------------    982
P. vivax (Sal-1)     LGKDDHDLEILESANSSKLKVEGRT-----------------------------------    1064
P. vivax (P01)       LGKDDHDLEILESANSSKLKVEGRT-----------------------------------    1057
                      ********       .  :  . .

P. falciparum        RDNSNKNRDNYNRNKDKNNTNRDNYNRYKDNNYYYNNSDNNNYNERKRYIRKKTYNKLSY    913
P. malariae          ------SGKSTSKST-----------------------------TSNQNNGKHVSHLGC    1316
P. ovale             ------VRQEYYRNGKKEG-NRRG---------------EGKYVGGKGGEKSRVNSELAY    1083
P. knowlesi          ------N---------------------------------------------KRIPKLGH    991
P. vivax (Sal-1)     ------S---------------------------------------------KRSPKLGH    1073
P. vivax (P01)       ------S---------------------------------------------KRSPKLGH    1066
                                                                         :   .*.

P. falciparum        FNLPSLKSIYNNKIKGNSEEF---------------------------SFDNELPEQTES    946
P. malariae          FTLPNVKSLDKHNNKQNEDNF--------------------------LSSPIKVQGDCEW    1350
P. ovale             FASPTVDELLKQSKGENSGHSADSADASSIANT------ANAVDVEGDDQSDSARDSGDW    1137
P. knowlesi          FVVPKLESVQKHSKEENKNAC---------IGGCSDDSSDDCSEDDESHVKHGIVGNSDL    1042
P. vivax (Sal-1)     FVVPKLESVEKHSTGGSGGSDGSDEDSDETTDAGSDGGSDGGSVGGGALAKHKIMGNSDW    1133
P. vivax (P01)       FVVPKLESVEKHSTGGSGGSDGSDEDSDETTDAGSDGGSDGGSVGGGALAKHKIMGNSDW    1126
                     *  *.:..: ::.    .                                      . :

P. falciparum        FPLNKPQDHEAFYNLKKHHTNVYEPNDEEKQNEQKL--KDQIKITSDILYKDIEENKNTE    1004
P. malariae          VPLKSPQNYEDFYNEKKRNTGLFEKSEESN-NENKL--SDDLNLKYNFFENGDIDNKKEE    1407
P. ovale             VPLKSPKNHEDFYNERKRYTNLFEDKTEKK-KKNVV--EDRLNLKYDFLEKEDKE-----    1189
P. knowlesi          IPLKSPPNHEDFYNAKKLQMNLFDHGGNKL-NEKEMEEKDGFILKFTSLEKGARANKAPE    1101
P. vivax (Sal-1)     IPLKSPPNHEDFYNAKKRHTNLLDHEGKQL-CEKKMDDSDCVSLKCAFLEKDARAKESGE    1192
P. vivax (P01)       IPLKSPPNHEDFYNAKKRHTNLLDHEGKQL-CEKKMDDSDCVSLKCAFLEKDARAKESGE    1185
                     .**:.* ::* *** :*   .: :   :.   :: :  .* . :.   : :

P. falciparum        DVLLIETITINNGTTSNTIENNKDSNKEAENSNTEQNDNNNNDNNNNINNNNNNNDNKEE    1064
P. malariae          DLYAK-NIHANDTSETDSLPLNGKTK----------K---------------KYHSNKS-    1440
P. ovale             ---------------------------------------------------NYNTDDT-    1196
P. knowlesi          TVEKNS-------DEGDNQKIDGGVTTG----GEDQA-----E-----------TENDE-    1133
P. vivax (Sal-1)     CDKEVVDKHTGDKEASDKQTGEGGET-D----GADQA-----E------SQNNVNESEE-    1235
P. vivax (P01)       CDKEVVDKHTGDKEASDKQTGEGGET-D----GADQA-----E------SQNNVNESEE-    1228
                                                                         ..
```

```
P. falciparum       DMNENNNNSKVTGDSVENINEQTNNNQYPNTEYNTIQRSINAKYLIFFFKNLHVWKTDLF    1124
P. malariae         -----------------NI-SSNSNSNNIGNNDSNVKKSIKAKYLIYFFKNIHVWKTQVY    1482
P. ovale            ----------------NLINSVKLKSRMRGTNAEKKKKVINAKYLIYFFKNIHVWKTSVY    1240
P. knowlesi         ----------------HSVDSLKLLNKMEKTYLHKKKKTISAKYLIYFFKNVHVWKTGEY    1177
P. vivax (Sal-1)    ----------------HSAESLKLQSKMGEPLSCKKKKAISAKYLIYFFKNVHVWKTGEY    1279
P. vivax (P01)      ----------------HSAESLKLQSKMGEPLSCKKKKAISAKYLIYFFKNVHVWKTGEY    1272
                                        . .  ..       . :: *.*****:****:*****  :

P. falciparum       CQNINYMNNYLNSIQYNKTLTFDINYDTNAVTIYFTDNITYTVKVNLEYLVFLLEKISLI    1184
P. malariae         CQNMNYINNLLENINYDEDIIFQENLEEDIVYVYFTSKLGVKYDIDVNYFIFLLQKISLI    1542
P. ovale            CQNMNYIDNLLKNINYNEDIVFQESVENGTVSLLFSSNLKDIYKIDMDYFIFLLQKISLI    1300
P. knowlesi         CQNMNYIDKFLKSINYKEEITFQEHLDQDSVVLQFTSSLKNIYKVDMDYFIFLLQKISLI    1237
P. vivax (Sal-1)    CQNVNYIDNWLKKINYNEEIIFQEQLDEDSVVLYFSSNLKDKYKLDMEYFIFLLQKISLI    1339
P. vivax (P01)      CQNVNYIDNWLKKINYNEEIIFQEQLDEDSVVLYFSSNLKDKYKLDMEYFIFLLQKISLI    1332
                    ***:**::: *:.*:*.: : *:   : . * : *:..:    .::::*::***:*****

P. falciparum       TFVEDLCSLFDTDKKRNYKNLTEFLENTERINTFVRNHMMLSNEQFINKNKYAKELAEIS    1244
P. malariae         MYVEDLCGIFQLDDVKQNQKLDRHLENTTNTQNFIEKHIIFFYEQYSNKNKYAKQLSNIS    1602
P. ovale            MYVEDLCGIFQLDEVKKNKKIDKYLEKKGNIHHFLEKHIIFSHEQYIKKNKYARKLSDVS    1360
P. knowlesi         MYVEDLCEIFQIEEMKQNKKIDKYIENEANIVNFIEKHLMFSHEQYSKKNKYAKELSIIS    1297
P. vivax (Sal-1)    MYVEDLCGIFQIDEMKQNKKIDKHIENPANIHNFMEKHILFSHEQYSKKNKYAKELSIIS    1399
P. vivax (P01)      MYVEDLCGIFQIDEMKQNKKIDKHIENPANIHNFMEKHILFSHEQYSKKNKYAKELSIIS    1392
                     :***** :*: :. :: ::: ..:*:  .   *:.:*:::  **: :*****::*: :*

P. falciparum       TSNLFYPKKDIILRSTPYNNIILDEKDIYQTIFIYMDDMLTEKMVNDTWITPYAFVVYSK    1304
P. malariae         TSTFFSSKKDIILNTESYNNIIFNEKDIYDVMFIYMEDVLTEKMVIDTWLTPYGFMLYKP    1662
P. ovale            TSNFFSSKKDIIFNPEPYNNVIFNEKEIYESLHTYMEDVLTEKMLNETWLTPYGFMLYSP    1420
P. knowlesi         TSNLFSSKKDIILNSQPYNNIVFNEKEIYESLFIYMEDVLTERMMNATWLTPYGFILYKQ    1357
P. vivax (Sal-1)    TSNFFSSKKDIILNSQPYNNIVFNEKEIYESLYVYMEDVLTERMINETWLTPYGFILCKP    1459
P. vivax (P01)      TSNFFSSKKDIILNSQPYNNIVFNEKEIYESLYVYMEDVLTERMINETWLTPYGFILCKP    1452
                    **.:*  *****:.   ***:::**:**:  :. **:*:***:*:  **:***.*:: .

P. falciparum       SKKD--MQGNNIKIEQNKNITKYSRSAIDKYVHYEYKRISENLNRFFMESNSNAPQFNEN    1362
P. malariae         NNDSGNNDNIKLSISKNENITKHSRNSIDKYLFYEYKKISNNILQYYDDLNSKIPQFKDN    1722
P. ovale            NK-A--NSKYKLKISQNVHITKYSRNAIDKYIYYEYKKISNNIIQYYEELNPKIHEYSDD    1477
P. knowlesi         PSKD--LNSYKLKIAQNEFITKFSRSAIDKYMYYEYRKISNNIVQHHVELSPKLGENLAE    1415
P. vivax (Sal-1)    TLSD--ANNYKLKISQNEFITKYSRSAIDKYMYYEYRKIGNNIVQHHLDLSPKLSEHLAE    1517
P. vivax (P01)      TLSD--ANNYKLKISQNEFITKYSRSAIDKYMYYEYRKIGNNIVQHHLDLSPKLSEHLAE    1510
                           .  ::.* :*  ***.**.:****:.***::*.:*: :.. : . :  :   :

P. falciparum       YKEYTIIIYNDNPSMPNIVLTTTINVFNVSFLQSIMEMLLNIKANQQFFSYKGKFIPINA    1422
P. malariae         LKEYKLVIYNDNPSMTNIVLTTTINVLNVALLQSILEVVLEIKANQQFFSYNGKFISLNT    1782
P. ovale            LKEYKLVIYNDNPSMTNIIIRTTVNVLNIAFLQSLLEVILDIRADQQFFSFKGKFLPVNA    1537
P. knowlesi         PKEYKLIIYNDNPSMTNIIITTSINVLNISFLHSLLEIILDIRATQQFFSYKGRFIPINA    1475
P. vivax (Sal-1)    LKEYRLIIYNDNPSMTNIIITTTVNVLNIAFLQSLLEVILDIRATQQFFSYKGRFIPINA    1577
P. vivax (P01)      LKEYRLIIYNDNPSMTNIIITTTVNVLNIAFLQSLLEVILDIRATQQFFSYKGRFIPINA    1570
                     *** ::******** **:: *::**:*:::*:*::*::*:*:* *****::*:*: :*:

P. falciparum       FITLENKINYIFFNYIPLENYVNNGDALDFRNP    1455
P. malariae         FIILDEGINYLFFNYIPNENHPALSNTGYA---    1812
P. ovale            FLILDEGINYLFFNYVPNENHINFTCQV-----    1565
P. knowlesi         FIILDEGVNYLFFNYAPNENHINEEA-------    1501
P. vivax (Sal-1)    FIILDEGVNYLFFNYVPNENHINYAA-------    1603
P. vivax (P01)      FIILDEGVNYLFFNYVPNENHINYAA-------    1596
                    *: *:: :**:**** * **:
```
