# Supplementary material for: Autocatalytic activation of a malarial egress protease is druggable and requires a protein cofactor
Source: EMBO J. 2021 May 1;40(11):e107226. doi: 10.15252/embj.2020107226 (PMC8167364; doi:10.15252/embj.2020107226)
Supplement: Supplementary file 2 — Expanded View Figures PDF [file EMBJ-40-e107226-s008.pdf]

Expanded View Figures

Figure EV1. Epitope tagging of SERA6 enables monitoring of SERA6 expression.

A Cas9-based approach for internal tagging and conditional disruption of the *SERA6* gene (PF3D7_0207500). Predicted catalytic triad residues (C, H, N), sgRNA targeting sites (break), *loxP* sites (arrowheads), recodonised sequences (hatched) and corresponding homology arms (regions linked by grey dotted lines) are shown. Primers used for diagnostic PCR to confirm gene editing and excision of floxed sequences are indicated (half-arrows) (see Table EV1 for primer sequences).

B Diagnostic PCR confirming modification of the *SERA6* locus and efficient DiCre-mediated disruption within the cycle of RAP treatment (cycle 0) (representative of 6 independent experiments).

C Western blots of DMSO- and RAP-treated *SERA6-mTAP:loxP* parasites showing successful epitope tagging and RAP-inducible ablation of SERA6-mTAP expression (representative of 2 independent experiments). Extracts of mature C2-arrested schizonts from the end of cycle 0 were probed with anti-HA then the blot stripped and reprobed with anti-AMA1 as a loading control. Red arrowhead, full-length SERA6-mTAP.

D Representative IFA images confirming RAP-induced loss of SERA6-mTAP expression (representative of 2 independent experiments). Mature C2-arrested cycle 0 schizonts were co-stained with anti-HA (red) and anti-MSP1 (mAb X509) (green). Merged signals include that of the DNA dye 4,6-diamidino-2-phenylindole (DAPI; blue). Scale bar, 5 μm.

E Replication of DMSO- and RAP-treated *SERA6-mTAP:loxP* parasites over three erythrocytic cycles. Parasitaemia values are averages from replicates in different blood sources. The similarity between DMSO-treated parasites and the parental B11 clone shows that the genetic modifications to generate *SERA6-mTAP:loxP* parasites do not affect parasite viability. Error bars, ± SD (B11: $n = 3$; DMSO- and RAP-treated *SERA6-mTAP:loxP*: $n = 6$ each).

F Western blot of DMSO- and RAP-treated *SERA6-mTAP:loxP* parasites showing host RBC β-spectrin cleavage during egress (red arrow) does not occur in the absence of SERA6-mTAP (reproducible in 2 independent experiments). Schizonts were sampled at the indicated times following removal of C2-arrest.

G Stills from time-lapse DIC microscopic examination of DMSO- and RAP-treated *SERA6-mTAP:loxP* cycle 0 schizonts at the indicated intervals following removal of C2-arrest (representative of 2 independent experiments). Note the defect in RBCM rupture and merozoite release in RAP-treated parasites despite normal PVM rupture (indicated by a sudden increased visibility and motility of merozoites). Scale bar, 5 μm.

Source data are available online for this figure.

© 2021 The Authors

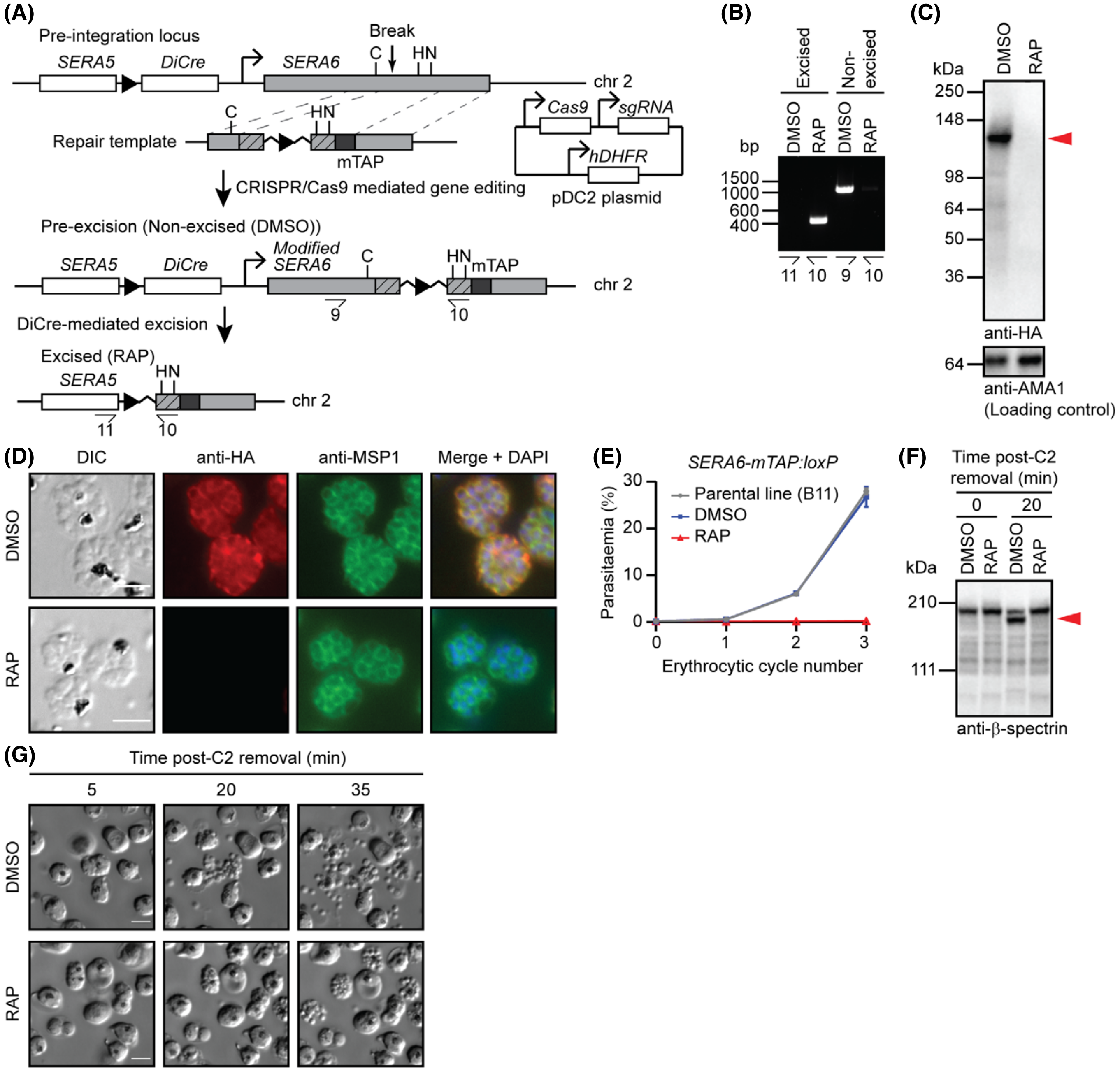
(A)
Pre-integration locus
SERA5
DiCre
SERA6
Break
C
HN
chr 2
Repair template
C
HN
mTAP
Cas9
sgRNA
hDHFR
pDC2 plasmid
CRISPR/Cas9 mediated gene editing
Pre-excision (Non-excised (DMSO))
Modified SERA6
mTAP
9
10
DiCre-mediated excision
Excised (RAP)
11
(B)
Excised
Non-excised
DMSO
RAP
bp
1500
1000
600
400
(C)
kDa
250
148
98
64
50
36
anti-HA
anti-AMA1
(Loading control)
(D)
DIC
anti-HA
anti-MSP1
Merge + DAPI
(E)
SERA6-mTAP:loxP
Parental line (B11)
Parasitaemia (%)
Erythrocytic cycle number
(F)
Time post-C2 removal (min)
0
20
210
111
anti-β-spectrin
(G)
5
35

Figure EV1.

```
   1 MICPIFFLYI INVLFTQYFI KCEGNKVTVI SHNNGHNDNL DVNKNGVISQ
  51 ENVFDTSESL NLPSNKKVGS DDLNTTTISF TVPDNLENEV KVVSSSESGK
 101 GATVSHTKVT SEGLSDTQPN VTQSVSSSTH TPGSLDSTMS TEQHSSVSQS
 151 SLPTESSSET LNKATVPEIP IQINSGLLKN YNGVKVTGSC GSYFRVYLVP
 201 HILIYALTKY SVIQLESLFN DNARIDVEHK GELQNKCSEG YHFKLVVYIT
 251 HNVLNLKWKT YKPNEESKSE DSDVRKYRIP KLERPFTSIQ VYTANSKAGV
 301 IETKNYNIRT DIPDTCDAIA TDCFLNGNVN IEKCFQCTLL VQKKDKSHEC
 351 FKYVSSEMKK KMNEIKVKAQ▼DDFNPNEYKL IESIDNILSK IYKKANKPFE
 401 ISKDLINLED LDYQFKNELL EYCKLLKKVD TSGTLEEYEL GNAEDIYNNL
 451 TRLLKSHSDE NIVTLQGKLR NTAICIKNVD EWILNKRGLT LPSESPSESS
 501 SKSDSYLNTF NDKDKNEDKD DMSKNSKEEF KNDDKENSDD QNNNDSNKKD
 551 DENNINNGDT NYVYDFDDDD YDNNSYEKDM YESPIKENKN GVIDLEKYGN
 601 QIKLKSPYFK NSKYCNYEYC NRWRDKTSCI SQIEVEEQGN CGLCWIFASK
 651 LHFETIRCMR GYGHFRSSAL YVANCSKRKP IDRCEEGSNP LEFLRILDEK
 701 KFLPLESNYP YSYTSAGNSC PKLPNSWTNL WGDTKLLFNK KVHRYIGNKG
 751 FISHETSYFK NNMDLFIDMV KREVQNKGSV IIYIKTQDVI GYDFNGKGVH
 801 SMCGDRTPDH AANIIGYGNY INKKGEKRSY WLIRNSWSYY WGDEGNFRVD
 851 MLGPKNCLYN FIHTVVFFKL DLGTIHVPKK KSWKKNYPYD VPDYAGYPYD
 901 VPDYAMGYPY DVPDYASAWS HPQFEKLENL YFQGVYFLRH NPDFMYSLYY
 951 NNYEPETSQD FESENDYDNA FVHGQ▼SNESD ETNKEGKNVH NSVEKKIQIL
1001 HILKHIKDSQ IKRGLVKYDN INETKDEHTC SRVNSQDAEK YEECKKFCLT
1051 KWNECKDHYS PGYCLTDLYK GEDCNFCYV
```

Figure EV2. The mTAP tag is "trimmed" in SERA6 PAP-mTAP.

Predicted primary sequence of SERA6-mTAP, showing peptides identified from SERA6 PAP-mTAP by mass spectrometry following trypsin or elastase digestion (highlighted in yellow). Predicted catalytic triad residues (bold, underlined) and SUB1 sites 1 and 2 (black arrowheads) are indicated. The mTAP tag sequence is indicated in bold, and its three components, the triple hemagglutinin (HA3) epitope tag (red), Strep-tag II sequence (blue) and tobacco etch virus (TEV) protease cleavage motif (green), are indicated. Loss of the Strep-tag II sequence by "C-terminal trimming" was corroborated by our inability to isolate PAP-mTAP using the Strep-Tactin® system which relies on the integrity of this sequence.

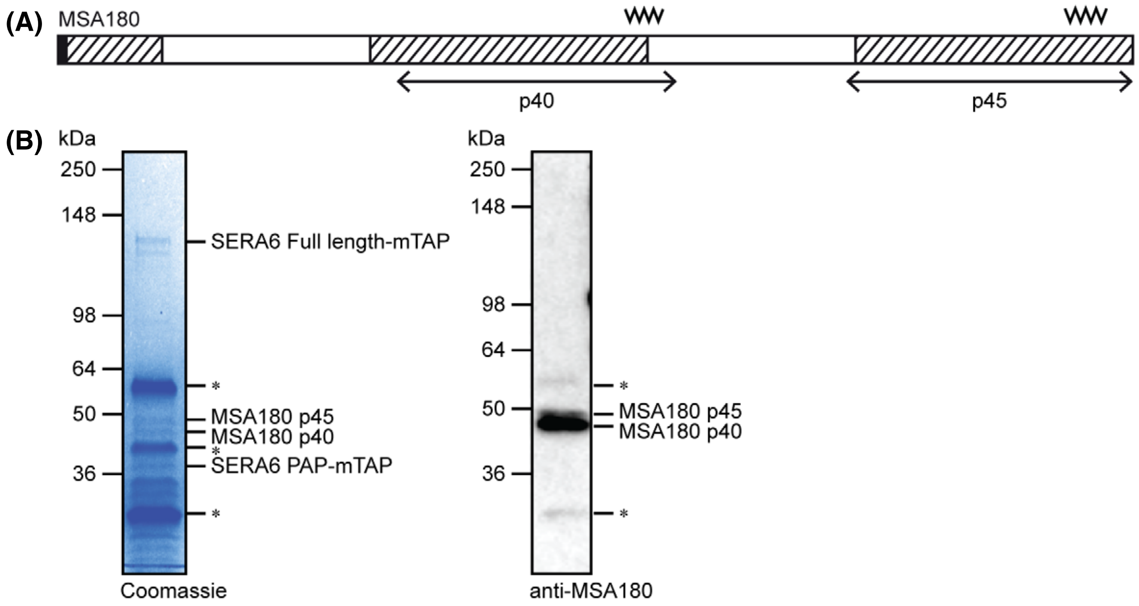

Figure EV3. Two distinct non-contiguous regions of MSA180 interact with SERA6 PAP, the mature form of SERA6.

A Schematic of MSA180. The predicted signal peptide (shaded black), positions of conserved regions (hatched; see Appendix Fig S2 for sequence alignments) and likely boundaries of the SERA6-interacting fragments (double-headed arrows) are shown. Positions of internal sequences used as synthetic peptides to raise polyclonal antibodies are indicated by zigzag lines.

B Left: Coomassie-stained SDS–PAGE gel showing immunoprecipitation products from SERA6-mTAP-expressing schizonts sampled at 20 min following removal of C2-arrest. Asterisks indicate antibody heavy and light chains. The unlabelled bands were also precipitated from extracts of parasites not expressing SERA6-mTAP (see Fig 1B) so are presumed to be non-specific. Right: Western blot of the same samples probed with polyclonal serum from a rabbit immunised with equal amounts of two MSA180-derived synthetic peptides (indicated in panel A). Despite being present in apparently similar stoichiometric amounts (as inferred from similar intensities on the Coomassie-stained gels), MSA180 p40 was more readily detected than MSA180 p45. Antibody heavy and light chains from the immunoprecipitation beads used were also weakly cross-reactive with the polyclonal rabbit serum (asterisks). Data shown were identical in 5 independent pull-down experiments.

Source data are available online for this figure.

© 2021 The Authors

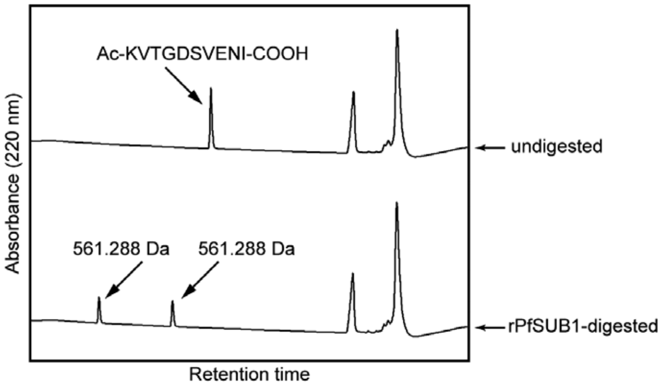

Figure EV4. A peptide spanning the putative SUB1 cleavage site ^{1074}KVTGD↓SVENI^{1083} in MSA180 is cleaved by recombinant *P. falciparum* SUB1 (rPfSUB1) as predicted.

Analytical RP-HPLC fractionation of N-acetylated synthetic peptide Ac-KVTGDSVENI, incubated without (top) or with (bottom) rPfSUB1 (reproducible in 2 independent experiments). Peaks corresponding to intact peptide and cleavage products were analysed by electrospray mass spectrometry. Masses of the cleavage products were identical (as expected if cleavage occurred at the central bond), hence identities of each peak could not be assigned.

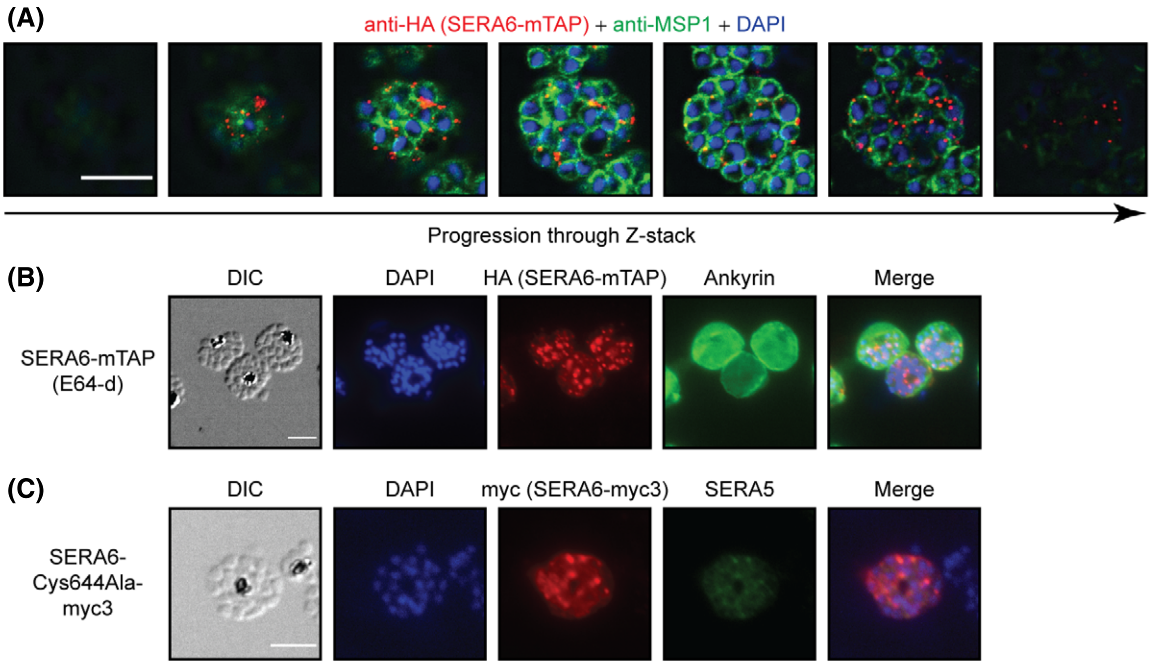

Figure EV5. SERA6 aggregates observed during egress are not associated with intracellular daughter merozoites and likely consist of the SERA6 p65 species.

A Z-stack series of a representative *SERA6-mTAP:loxP* schizont examined 5 min following removal of C2-arrest, fixed and co-stained with anti-HA (red) and anti-MSP1 (mAb X509) (green), imaged on an Olympus SpinSR10. Scale bar, 5 µm.

B Representative IFA images showing formation of SERA6 foci in *SERA6-mTAP:loxP* schizonts in the presence of E64-d, which arrests SERA6 maturation at the p65 stage and blocks egress (representative of 2 independent experiments). Parasites were co-stained with DAPI (blue), anti-HA (red) and anti-ankyrin (green). Scale bar, 5 µm.

C Representative IFA images showing formation of SERA6 foci in a RAP-treated *SERA6-Cys644Ala-myc3 [SERA6-mTAP:loxP]* schizont. Maturation of SERA6 in these parasites is arrested at the p65 step. Parasites were co-stained with DAPI (blue), anti-myc (red) and anti-SERA5 (green). Scale bar, 5 µm.

 © 2021 The Authors
