# Supplementary material for: Autocatalytic activation of a malarial egress protease is druggable and requires a protein cofactor
Source: EMBO J. 2021 May 1;40(11):e107226. doi: 10.15252/embj.2020107226 (PMC8167364; doi:10.15252/embj.2020107226)
Supplement: Supplementary file 3 — Table EV1 [file EMBJ-40-e107226-s002.pdf]

Table EV1. Oligonucleotide primers used in this study

Primer #	Sequence (5´-3´)
1	GGATAGTAATAAGGAAGCAGAAAATTCTAACAC
2	TCATCTAATATAATATTGTTATAAGGTGTGGA
3	CAATCCATATTATATGTTCAGATTAACTA
4	CGTCTACATAAAATTTACAAGTACAATTTAATGCTATGGT
5	GTGTTAGGTAAAATACATAACATTCTTAAAGGTAAATA
6	GTCGTAAGGGTAGCCCATGGC
7	CATTAGTAGATAATAAGGATTATAACAATAGTGAAG
8	CGTAACCTTTTCACTATGAATTTTG
9	GGATAAAAACGAAGATAAGGATGATATGAG
10	CTTCGGCCCAAGCATATCCA
11	GAATGCTATTTCTGCTACGTG
