# Supplementary figures and images for: Autocatalytic activation of a malarial egress protease is druggable and requires a protein cofactor
Source: EMBO J. 2021 May 1;40(11):e107226. doi: 10.15252/embj.2020107226 (PMC8167364; doi:10.15252/embj.2020107226)

Appendix Figure S1

A

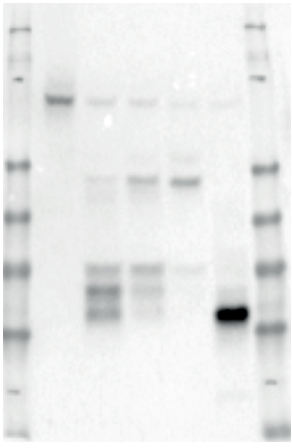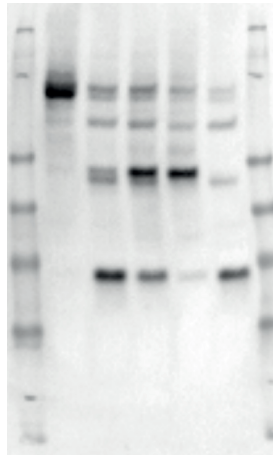

B

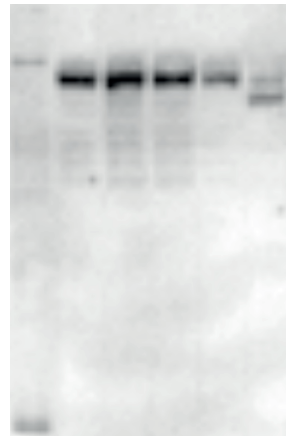

Supplement: Supplementary file 4 — Source Data for Expanded View and Appendix [file EMBJ-40-e107226-s003.zip › EMBOJ-2020-107226R1-Figure_S1_Source_Data-sd.pdf]

Figure 1

A

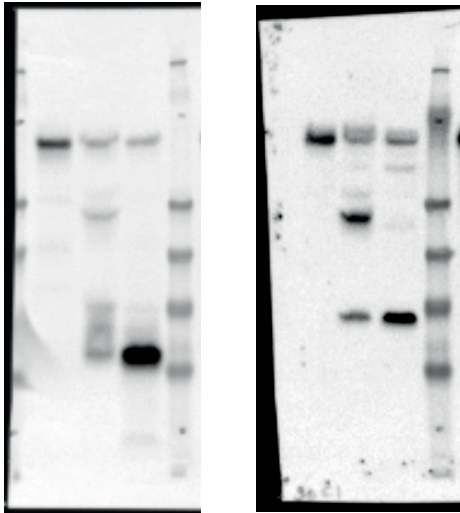

B

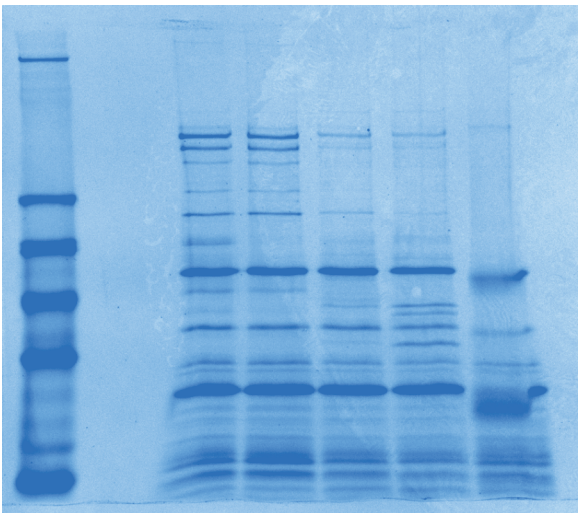

Supplement: Supplementary file 6 — Source Data for Figure 1 [file EMBJ-40-e107226-s005.pdf]

B

Figure 2

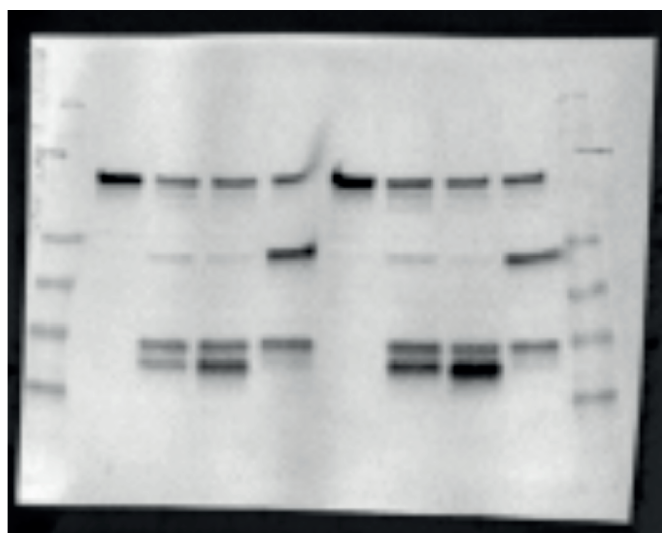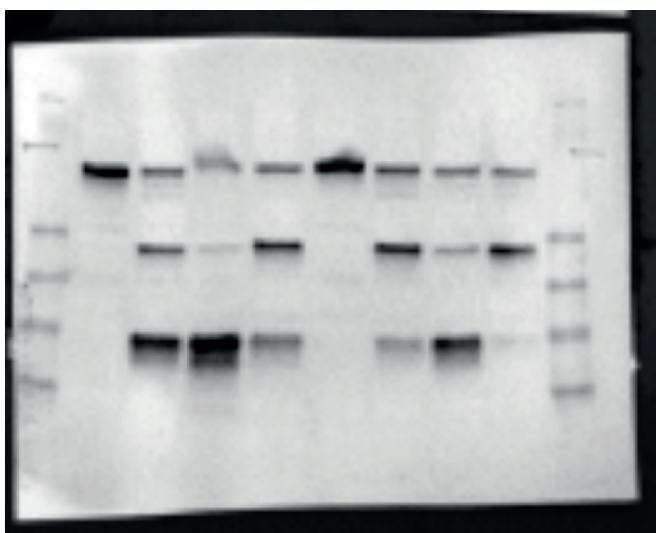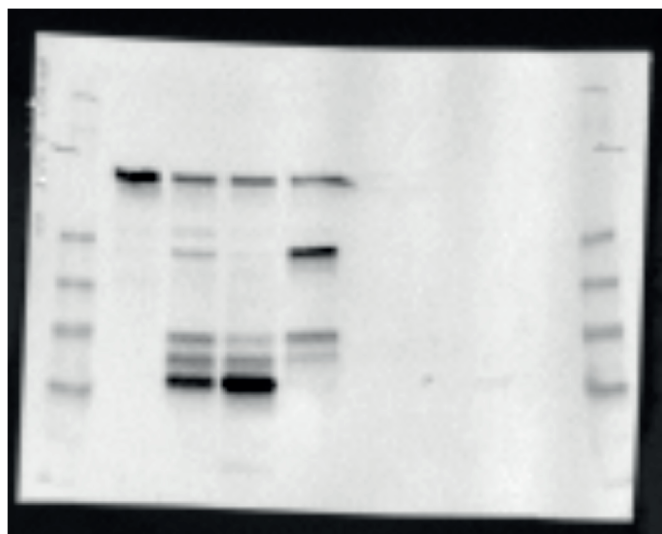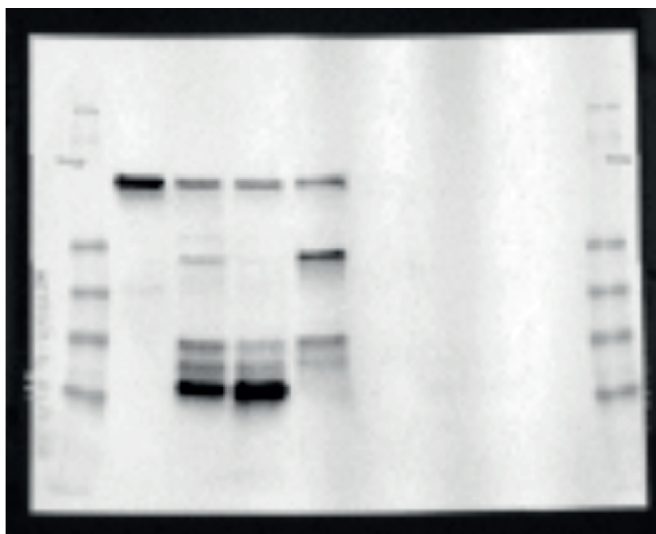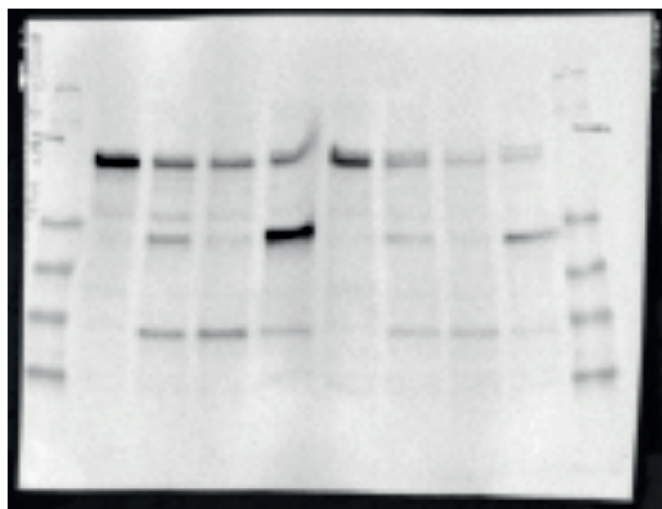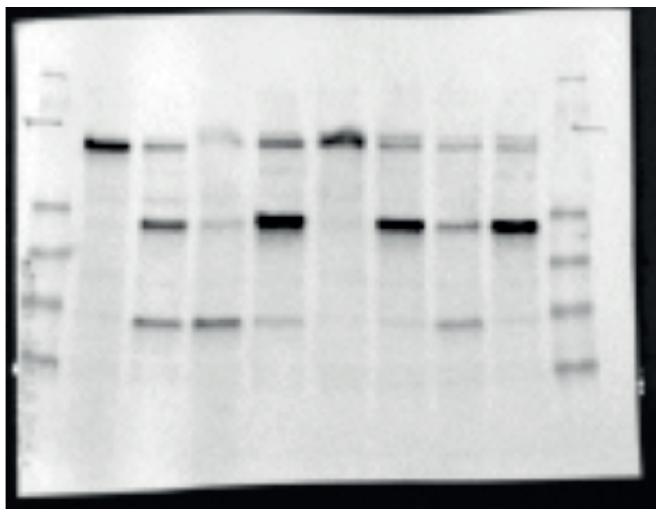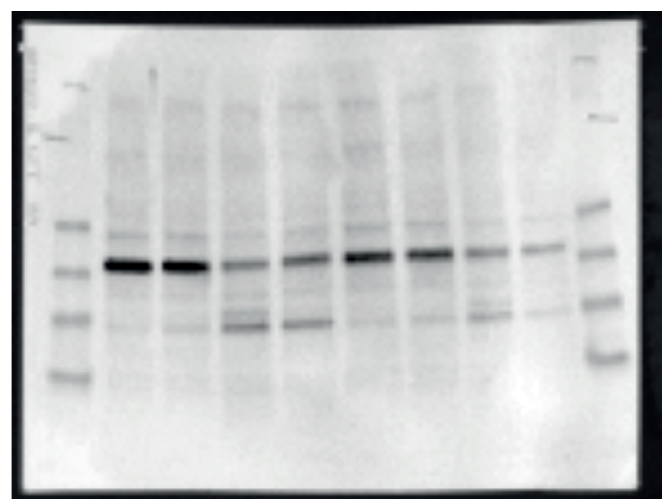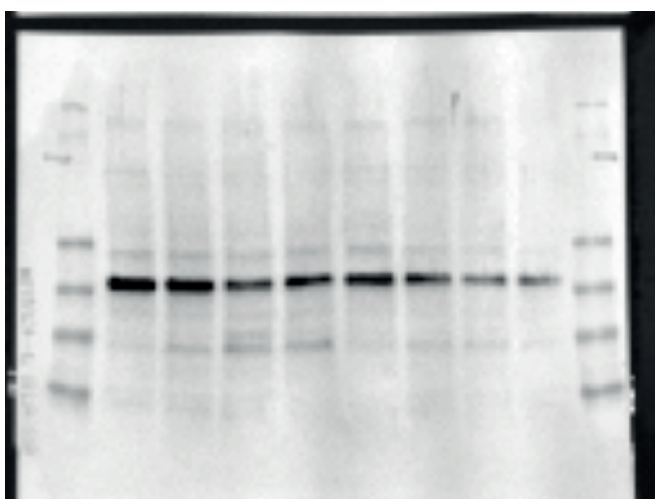

Figure 2

C

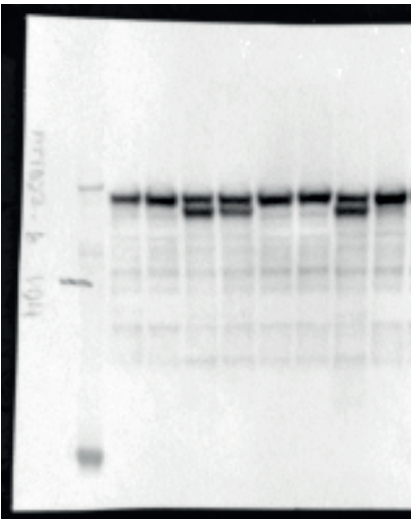

Supplement: Supplementary file 7 — Source Data for Figure 2 [file EMBJ-40-e107226-s004.pdf]

Figure 3

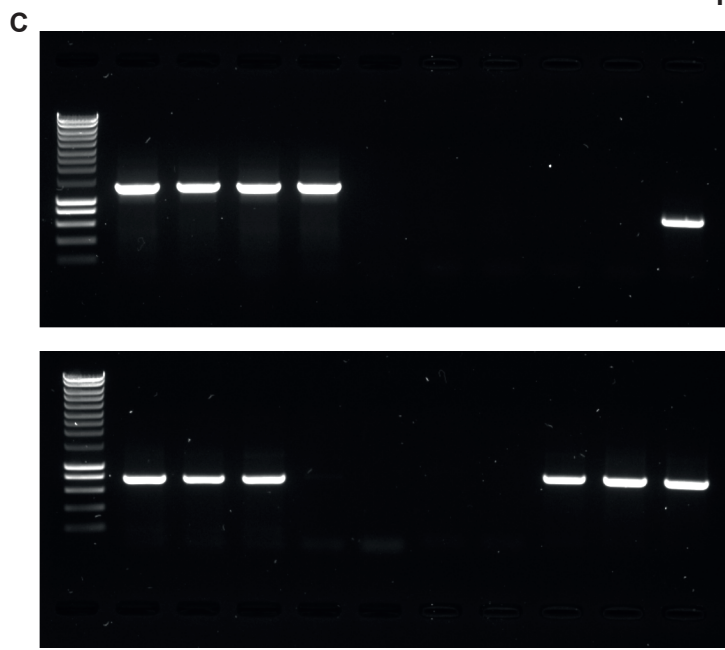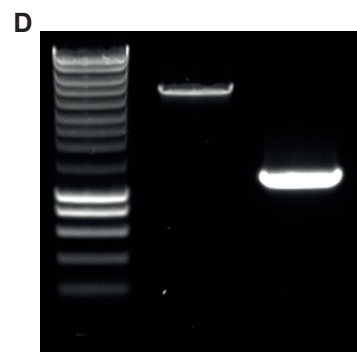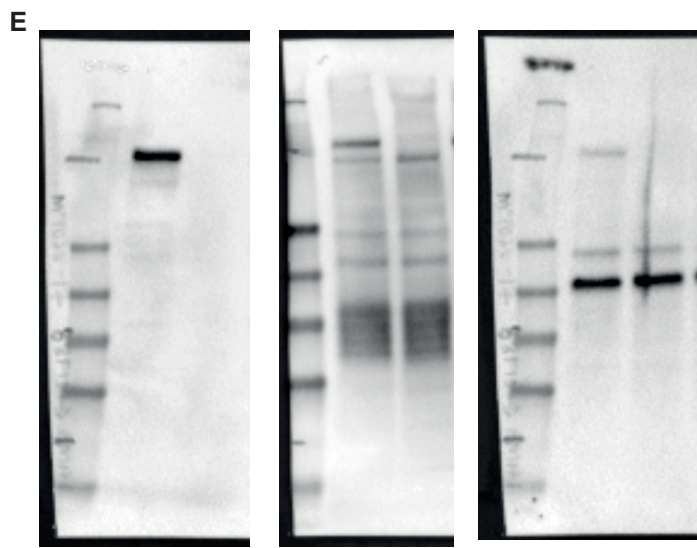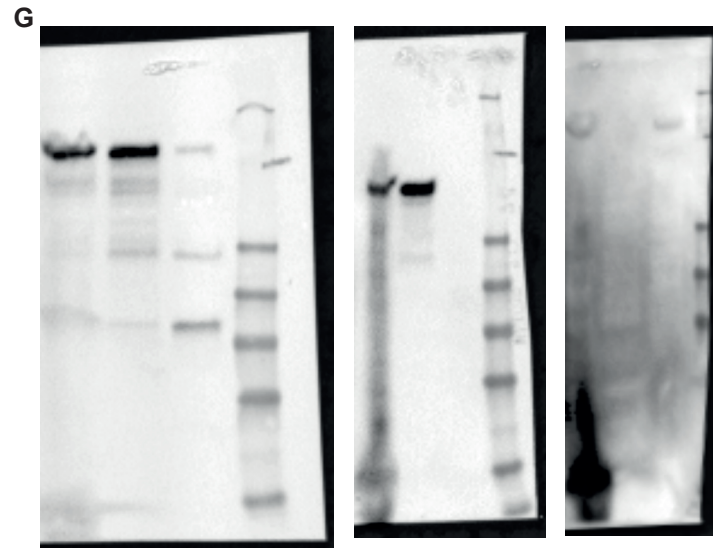

Supplement: Supplementary file 8 — Source Data for Figure 3 [file EMBJ-40-e107226-s007.pdf]

Figure 4

C

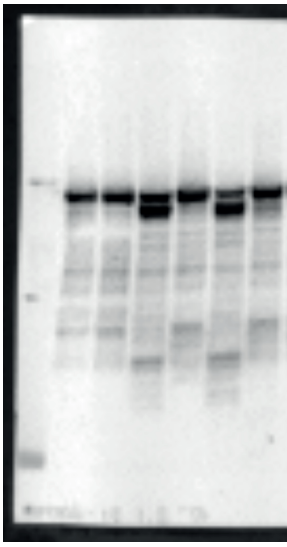

D

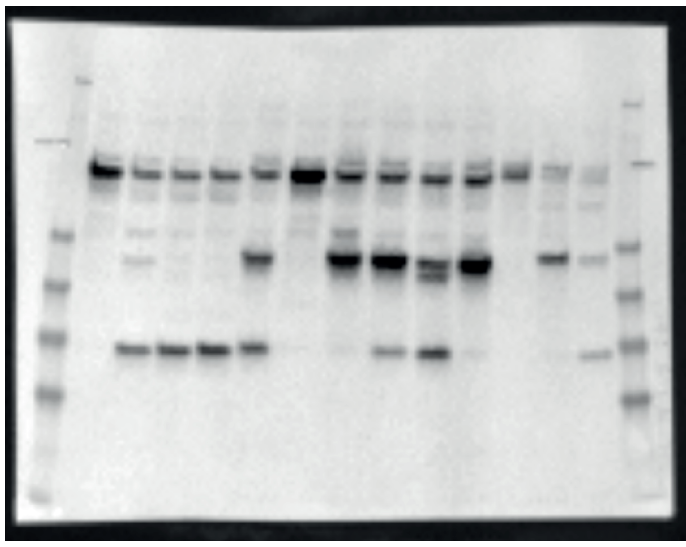

Supplement: Supplementary file 9 — Source Data for Figure 4 [file EMBJ-40-e107226-s006.pdf]

Figure 6

C

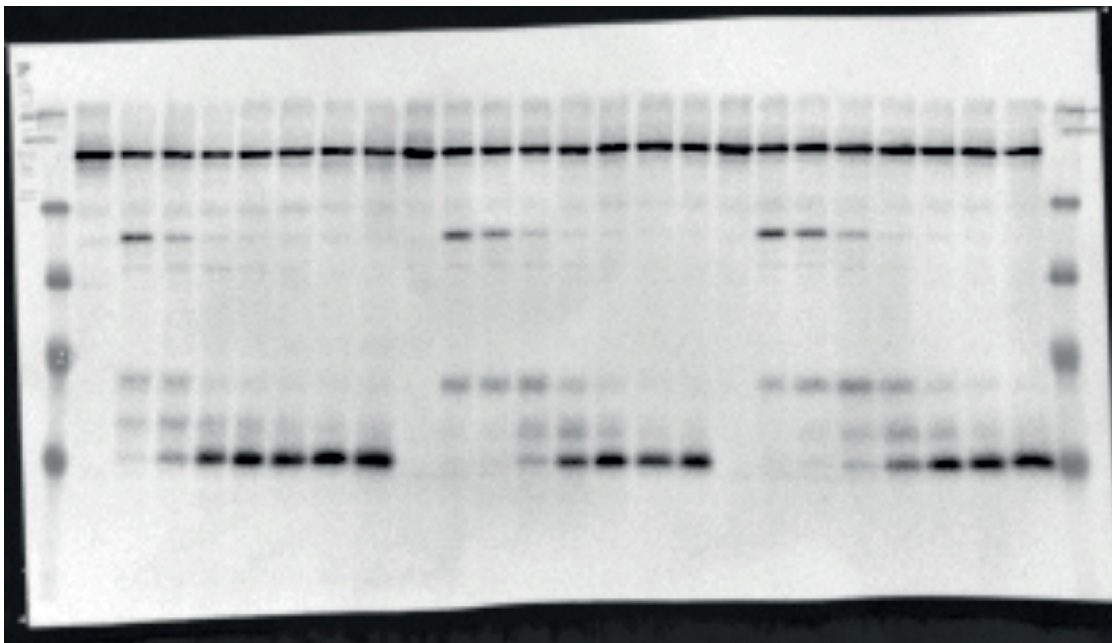

D

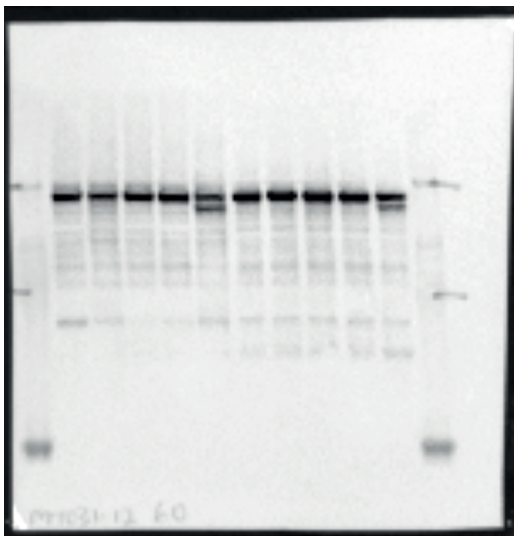

Supplement: Supplementary file 10 — Source Data for Figure 6 [file EMBJ-40-e107226-s010.pdf]
