# Supplementary material for: Autocatalytic activation of a malarial egress protease is druggable and requires a protein cofactor
Source: EMBO J. 2021 May 1;40(11):e107226. doi: 10.15252/embj.2020107226 (PMC8167364; doi:10.15252/embj.2020107226)

Figure EV1

B

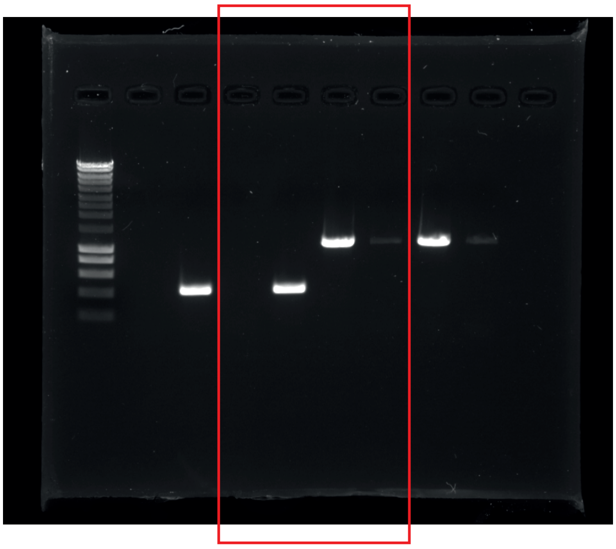

C

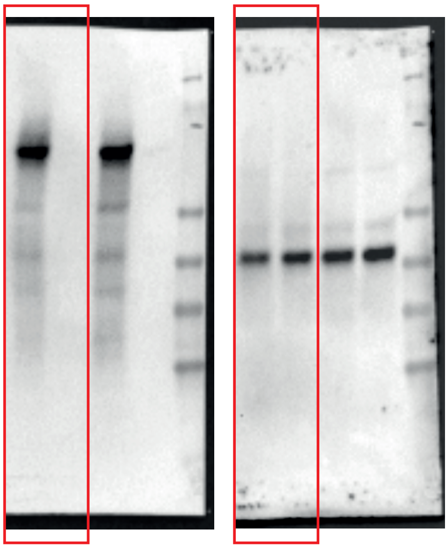

F

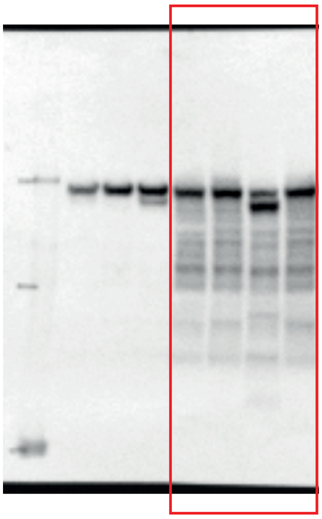

Supplement: Supplementary file 4 — Source Data for Expanded View and Appendix [file EMBJ-40-e107226-s003.zip › EMBOJ-2020-107226R1-Figure_EV_Source_Data.zip!/EMBOJ-2020-107226R1-Figure_EV1_Source_Data-sd.pdf]
